# Rider sitting position widens lumbar intervertebral distance: a prospective observational study

**DOI:** 10.1016/j.bjane.2021.03.010

**Published:** 2021-04-19

**Authors:** Melike Korkmaz Toker, Basak Altiparmak, Ali Ihsan Uysal, Mustafa Turan, Semra Gumus Demirbilek

**Affiliations:** a Mugla S..tk.. Kocman University, Department of Anesthesiology and Reanimation, Mugla, Turkey; b Mugla S..tk.. Kocman University Research and Training Hospital, Department of Anesthesiology and Reanimation, Mugla, Turkey; c The Health of Ministry of Republic of Turkey Ankara City Hospital, Ankara, Turkey

**Keywords:** Anesthesia, spinal, Ultrasonography, Spinal column

## Abstract

**Background:**

Reduced lumbar lordosis may make the process of identifying the intervertebral distance easier. The primary aim of this study was to measure the L3...L4 intervertebral space in the same patients undergoing spinal anesthesia in three different sitting positions, including the classic sitting position (CSP), hamstring stretch position (HSP) and rider sitting position (RSP). The secondary aim was to compare ultrasonographic measurements of the depth of the ligamentum flavum and intrathecal space in these three defined positions.

**Methods:**

This study is a single-blinded, prospective, randomized study. Ninety patients were included in final analysis. the patients were positioned on the operating table in three different positions to perform ultrasonographic measurements of the spinal canal. The intervertebral distance (IVD), the distance between the skin and the ligamentum flavum (DBSLF) and the intrathecal space (IS) were measured in the L3...L4 intervertebral space in three different positions.

**Results:**

The RSP produced the largest mean distance between the spinous processes. The RSP yielded a significantly larger IVD than did the CSP (*p* < 0.001) and HSP (*p* < 0.001). The DBSP was larger in the CSP than in the HSP (*p* = 0.001). The DBSLF was significantly larger in the RSP than in the HSP (*p* = 0.009).

**Conclusions:**

Positioning the patient in the RSP significantly increased the intervertebral distance between L3...L4 vertebrae compared to the CSP and HSP, suggesting easier performance of lumbar neuraxial block.

## Introduction

Lumbar spinal or epidural anesthesia is frequently administered in various surgeries to provide anesthesia and postoperative analgesia. The most important factor affecting success during spinal and epidural interventions is the patient...s positioning.^1^ Reduced lumbar lordosis may facilitate the palpation of vertebral spinous processes and identification of intervertebral distance.^2^, ^3^

Anesthesia textbooks outlined two regular patient positions as the lateral decubitus position and the sitting position^4^ however there are several trials comparing different modified sitting positions.^1^, ^2^, ^5^ Tashayod et al. described a modified sitting position named as hamstring stretch position and Manggala et al. described crossed leg sitting position in Asian population.^6^ All authors describing these modified sitting positions have the same purpose of achieving the optimal flexed position to reduce the lumbar lordosis and open the intervertebral space. In addition to these positions described in the literature, another sitting position that we call ..úthe rider sitting position...... is commonly applied during spinal epidural anesthesia in our clinic. This position has not been previously described in the literature. However, anecdotal evidence in our experience suggested that induction of spinal anesthesia with the patients positioned on the table like they were riding a horse with the knees flexed 90 degrees and the feet swinging freely, legs placed on the table, make spinal puncture easier.

The use of ultrasonography (US) to increase success during neuraxial block applications has gained popularity among anesthesiologists in recent years. Ultrasound imaging for vertebral canal can display the spine of vertebra, the optimal needle insertion point and can identify the soft tissue acoustic window between vertebral laminas and also can measure the intervertebral distance and the extent of the ligamentum flavum.^7^, ^8^, ^9^ The optimal position for lumbar punctures to figure out maximal intervertebral distance has been assessed in pediatrics and adults by using ultrasonography and radiography.^10^, ^11^, ^12^ However, none of these studies include the position defined as the rider sitting position in our clinic.

The primary aim of this study was to measure the acoustic target (defined as the visualized L3...L4 intervertebral space) of the same patients undergoing spinal anesthesia in three different sitting positions, defined as classical sitting position (CSP), hamstring stretch position (HSP), and rider sitting position (RSP). The secondary aim was to compare ultrasonographic measurements of the depth of ligamentum flavum and intrathecal space at these three defined positions.

## Methods

This observational study was approved by Mugla Sitki Kocman University Training and Research Hospital Biomedical Research Ethics Committee on September 17, 2019 and registered at anzctr.org.au (Trial ID: ACTRN12619001753145) and conducted in accordance with the current Declaration of Helsinki. The study adheres to CONSORT guidelines. After obtaining written informed consents from the participants, patients who underwent surgery under spinal anesthesia were considered for the study. Patients between 18...45 years old with American Society of Anesthesiologists (ASA) physical status I...III and scheduled for spinal anesthesia were prospectively included in the study. The exclusion criteria were patients with history of previous lumbar vertebral surgery, significant spinal anatomical abnormalities, allergy to ultrasound gel, those whose body mass index (BMI) > 30 kg.m^-2^ or those who presented a language barrier. Subjects who did not want to participate were excluded. The age, height, weight, and BMI of all the participants were recorded. Participants were sequentially enrolled to the study.

**Figure 1:**
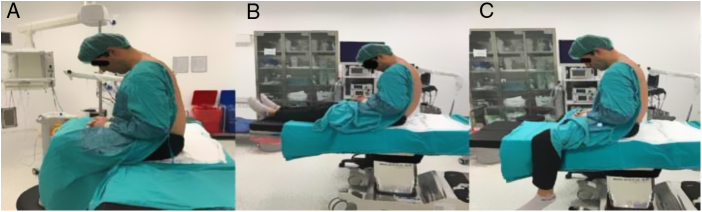
**A**, The volunteer at the classical sitting position; **B**, The volunteer at the hamstring stretch position; **C**, The volunteer at the rider sitting position.

**Figure 2:**
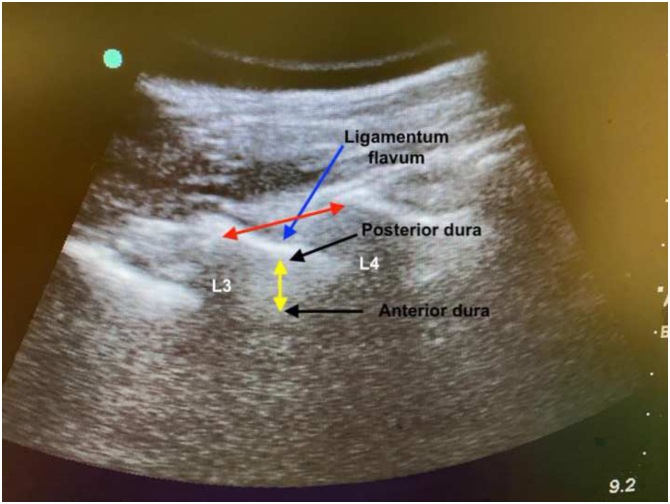
Paramedian longitudinal oblique ultrasonographic view of the lumbar spine at the level of the lamina showing the L3...4 and L4...5 interlaminar spaces. Red arrow: intervertebral distance; Yellow arrow: intrathecal space; L3: Lamina of L3 vertebrae; L4: Lamina of L4 vertebrae.

After the enrolled patients arrived at the operating room, standard monitoring procedures per ASA were applied. Before the administration of spinal anesthesia, patients were positioned on the operating table in three different positions, respectively. All patients were approached to twist forward and curve out their back maximally. The patients were told to sit in the CSP for the measurements of lumbar spinal canal, then move to the HSP when the measurement was over, and sit in the RSP after the 2nd measurement of spinal canal. At the CSP, the knees were flexed approximately 90 degrees, the hip was on abduction, and the feet were on a stool support (Fig. 1A). At the HSP, the patients were seated with the legs totally supported by the operating table and were asked for knee extension and hip adduction (Fig. 1B). At the RSP, patients were positioned on the table like they were riding a horse with the knees were flexed 90 degrees and the feet were swinging freely (Fig. 1C). For every position on the same patient, ultrasonographic measurements of spinal canal were performed by the same anesthesiologist (M.K.T) with at least 50 patient experience in ultrasonography in neuraxial blocks and images were recorded. The ultrasonographic evaluation was performed with a curvilinear 5 2 MHz US probe (SonoSite MTurbo; FUJIFILM SonoSite, Bothell, WA). In all three positions, curvilinear ultrasonography probe was applied on the longitudinal paramedian position, 1...2 cm lateral to the spinous process, initially articular process view had been obtained. Then the probe was slightly tilted medially to beam the lamina of L3, L4, and L5 vertebrae, saw-like image of the lumbar vertebra was recognized as Chin et al. defined in their study.^13^ First the intervertebral spaces and then the targeted L3...L4 intervertebral space were identified. The ligamentum flavum (LF) was determined as an echogenic structure inside the intervertebral space. These displays were recorded to the ultrasound own memory. All recorded ultrasonography images evaluated by a different anesthesiologist (B.A.) who was blinded to the positions. The intervertebral distance between the L3...L4 laminae (IVD), the distance between the skin and the ligamentum flavum (DBSLF), and the distance between anterior and posterior dura defined as intrathecal space (IS) were measured in the paramedian sagittal plane in L3...L4 intervertebral place using in built-in calipers (Fig. 2). The specific point on the spinous process was determined by a method that was described in a previous study.^14^ The acoustic shadows of the L3 and L4 lamina was determined. The IVD was measured as the distance between the apexes of the acoustic shadows of the L3 and L4 lamina. The IVD, DBSLF and the diameter of IS were recorded in three positions for every participant (three measurement for every position), thus there were 9 measurements for every subject.

## Statistical analysis

The sample size of the study was calculated using the G*Power program (v3.1.9.2). We conducted a pilot study with 15 patients in our clinic. According to this pilot study, the mean difference of 0.15 cm with the standard deviation 0.02 cm between CSP and RSP in the IVD measurement accepted as clinically significant. Assuming ..-error = 0.01 (two-tailed), and ..-error = 0.10 with a power of 90%, at least 76 patients were needed in total. Considering a 20% drop-out, we decided to include 92 patients in total.

Suitability for the normal distribution of the measured variables of IVD, DBSLF, and IS were examined using Shapiro-Wilk test. For parametric distributions, data were detailed with mean .. standard deviation and analyzed using analysis of variance test. Taking steps further with Anova, post-hoc tests were performed using least significant difference for pairwise comparisons. Analyses were performed using Statistical Package for Social Science (SPSS) version 25 (made by SPSS Incorporated, located in Chicago, Illinois, USA). A *p-*value of < 0.05 was considered as statistically significant.

## Results

**Figure 3:**
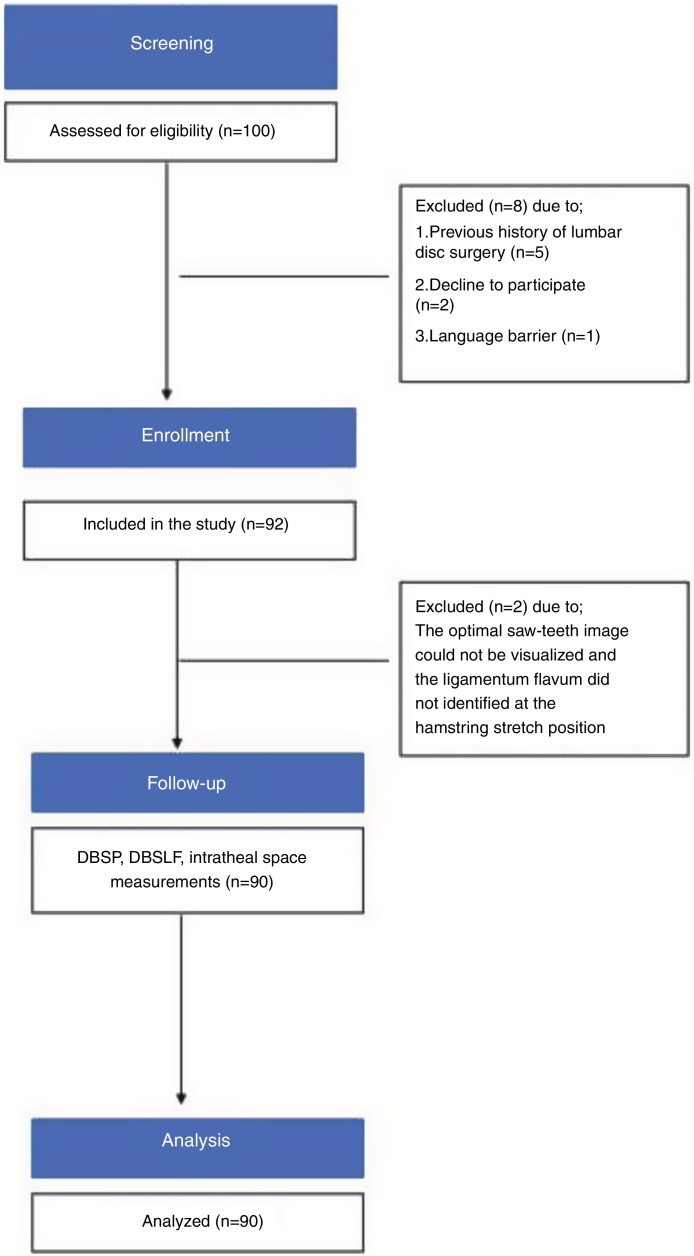
Flowchart of the study.

The statistical analysis included 90 patients (Fig. 3). Patient recruitment and enrollment was made in December 2019. The mean .. standard deviation (SD) age of the participants was 44.73 .. 11.5 years, their mean height was 169.8 .. 7.2 cm, their mean weight was 74.1 .. 10 kg and their mean BMI was 25.6 .. 2.3 kg.m^-2^ (Table 1).

**Table 1:** Patient demographics.

Demographics	
**Age (years)**	44.73 .. 11.5
**Weight (kg)**	74,1 .. 10
**Height (cm)**	169,8 .. 7,2
**BMI**	25,6 .. 2,3
**Gender**	
Female	43 (47.8%)
Male	47 (52.2%)
**ASA (I/II/III)**	27/56/7 (30%/62%/8%)

Data are expressed as the mean .. SD or as the number and percentage of patients.

BMI, Body Mass Index; ASA, American Society of Anesthesiologists physical status.

The L3...L4 intervertebral space of 90 participants were identified in three different positions. The mean .. standard deviation and median of IVD, DBSLF, and IS measurement for each position are shown in Table 2.

**Table 2:** Means and Medians of the intervertebral distance, the distance between skin to ligamentum flavum, intrathecal space in each position.

Measurements	Classical sitting position	Hamstring stretch position	Rider sitting position
**IVD (cm)**			
Mean .. SD	3,39 .. 0,37	3,19 .. 0,36	3,61 .. 0,41
Median [IQR]	3.35 [3.14...3.65]	3.15 [2.94...3.5]	3.58 [3.25...3.87]
**DBSLF (cm)**			
Mean .. SD	5,04 .. 0,47	5,14 .. 0,46	4,95 .. 0,5
Median [IQR]	5.17 [4.63...5.45]	5.3 [4.7...5.54]	5.1 [4.47...5.36]
**IS (cm)**			
Mean .. SD	1,3 .. 0,12	1,29 .. 0,11	1,45 .. 0,13
Median [IQR]	1.28 [1.22...1.35]	1.28 [1.23...1.33]	1.41 [1.37...1.49]

IVD, intervertebral distance; DBSLF, difference between skin to ligamentum flavum; IS, Intrathecal space; SD, standard deviation.

The mean differences of the IVD, DBSLF and IS between three positions was calculated with a 95% confidence interval. The mean differences were figured out with pairwise comparisons providing adjusted *p-*values (Table 3). The RSP position produced the largest mean distance between spinous processes. The RSP significantly increased the IVD comparing to CSP (*p* < 0.00) and HSP (*p* < 0.001). Also, the DBSP was higher at the CSP comparing to HSP (*p* = 0.001).

**Table 3:** Pairwise comparisons of the measured parameters.

			95% CI		
Parameter		Mean Difference	Lower	Upper	*p*^a^
**IVD (cm)**	CSP minus HSP	0.20	0.09	0.31	0.001
	CSP minus RSP	...0.21	...0.33	...0.10	0.000
	HSP minus RSP	...0.41	...0.53	...0.30	0.000
**DBSLF (cm)**	CSP minus HSP	...0.10	...0.24	0.04	0.159
	CSP minus RSP	0.09	...0.05	0.23	0.223
	HSP minus RSP	0.19	0.05	0.33	0.009
**IS (cm)**	CSP minus HSP	0.00	...0.24	0.04	0.159
	CSP minus RSP	...0.15	...0.05	0.23	0.223
	HSP minus RSP	...0.16	0.05	0.33	0.009

CI, confidence interval; IVD, intervertebral distance; DBSLF, difference between skin to ligamantum flavum; IS, intrathecal space.

Anova models for IVD, DBSLF, IS are at *p* = 0.000, *p* = 0.032, and *p* = 0.000, respectively.

a Obtained with posthoc test with LSD.

When the DBSLF measurements were taken into consideration, the DBSLF significantly increased at the RSP comparing to HSP (*p* = 0.009). Although the RSP decreased the DBSLF comparing to CSP, the difference of the means was not significant (*p* = 0.223). The RSP resulted in the largest mean IS comparing to CSP (*p* = 0.223) and HSP (*p* = 0.009).

## Discussion

In this study we measured the intervertebral space at L3...L4, the distance between the skin to ligamentum flavum, and the intrathecal space in different positions with ultrasonography. We demonstrated that with the rider sitting position, the intervertebral distance at L3...L4 interlaminar space identified with the preinsertion lumbar ultrasonography increased, and this extend was significant compared to CSP and HSP. The distance between the skin and the ligamentum was similar in both the CSP and the RSP. This study also demonstrated the benefits of the RSP compared to HSP by achieving the decreased DBSLF and increased IS.

Sandoval et al. compared the three different positions for lumbar puncture to identify the widest interspinous distance with US at the emergency department.^10^ The mean of intervertebral L4...L5 space of their 16 volunteers were reported between 1.91 cm to 2.02 cm in three different positions. In our study the mean of the L3...L4 intervertebral space in three different positions were approximately 50...60% wider comparing to their results. We assumed that performing our measurements at the longitudinal paramedian approach instead of sagittal approach and using the apexes of the acoustic shadows of two lamina as a reference point caused this difference. They reported that the sitting and the feet supported position produced the widest interspinous space. In their study, sitting and the feet supported position was the same as classical sitting position in our study. However, the compared sitting and the feet unsupported position was not the same as RSP in our study. At the RSP, the hips were abducted on the operating table in our study and we believe that the hip abduction in that position makes the patient more comfortable for reducing the lumbar lordosis.

Abo et al. determined the different sitting and lateral recumbent positions of children under 12 years at which position the intervertebral space maximized for lumbar punctures.^12^ They included 28 patients and recommended the sitting position with flexed hips for maximally increased interspinous space. In their study, they classified sitting positions as sitting or sitting with maximal flexion of the hips and at the sitting with flexed position they measured the maximal interspinous space. The sitting with maximal flexion of the hips matched the CSP in our study but they did not define the RSP as our study because the sitting position did not match the RSP in our study. When we applied the RSP, we told our patients to take their waist out like a scared cat. So, the sitting position with the maximal flexion of the hips may match the RSP in our study.

The comparison of spinal needle bone contact by reducing lumbar lordosis with different positions had been the objective of several studies. Fisher et al. determined scheduled the number of needle bone contact was similar in both traditional sitting position (TSP) and HSP groups on 406 pregnant patients.^2^ Similarly, Mohammadi et al. compared the number of spinal needle-bone contacts and ease of needle insertion at TSP, HSP and squatting position (SP).^1^ They found no statistical difference between the TSP, HSP, and SP regarding. However, there were no studies determining the measurements of intervertebral space with ultrasonography before spinal anesthesia and comparing the differences between different positions. Also, to the best of our knowledge this is the first study to evaluate the effect of rider sitting position to intervertebral space. In our study, the IVD at the CSP and HSP was significant and greater at the CSP however at the above studies there were no differences of the number of needle bone contacts and the ease of needle insertion at the two-position knowing the TSP matched the CSP in our study. Although according to our study the IVD widen at the CSP as 0.20 cm, because experienced anesthetists perform neuraxial anesthesia in the study of both Fisher^11^ and Mohammadi^1^ may have led to this result.

When it is determined that the greatest distance between L3...L4 laminae reached at the RSP, the close look to the hip during three positions is essential.

In our study, the common point of all three positions was hip flexion, while the hip was adducted in the CSP and HSP, whereas the hip was abducted in the RSP. Although we could not find any evidence about the hip abduction reduces lumbar lordosis, in our opinion at hip abducted position patients flexed their vertebral column more easily.

Previous studies demonstrated a good correlation between US guided skin to ligamentum flavum distance and definite needle depth.^8^, ^15^, ^16^ In the current study, the DBSLF which is important in overweight, obese, and pregnant patients did not differ significantly between CSP and RSP. Although at the RSP the DBSLF reached to the shortest measure, it was only significant when compared with the HSP.

The intrathecal space demonstrated and measured as an anechoic space between the posterior and anterior complexes of the intervertebral space. The RSP and CSP reached the greatest diameter of IS compared to the HSP. The local anesthetic drugs are administered to the IS during spinal anesthesia. The engorgement of IS would be practical in dehydrated patient for free flow of cerebrospinal fluid.

Grau et al. analyzed the transverse, median longitudinal and paramedian longitudinal approaches of vertebral US and compared the quality of monitoring and concluded their study as the paramedian longitudinal window was excellent.^17^ We performed US imaging through the paramedian longitudinal approach in our patients.

The strength of our study was that the recorded images were evaluated by a blinded anesthesiologist. The single blinding supported our results.

One limitation of this study was that it evaluated only patients with normal BMI or overweight. So, the results may not be applicable to the obese patients. Another limitation was that we did not compare any success rate of neuraxial anesthesia, number of needle bone contacts, or easiness of neuraxial anesthesia. Thus, clinical studies with these positions will be required for confirmation.

## Conclusion

In conclusion, positioning the patient in the RSP significantly increased the L3...L4 intervertebral distance compared to the CSP and HSP, suggesting easier performance of lumbar neuraxial block.

## Conflicts of interest

The authors declare no conflicts of interest.

## References

[1] Soltani Mohammadi S., Piri M., Khajehnasiri A. (2017). Comparing three different modified sitting positions for ease of spinal needle insertion in patients undergoing spinal anesthesia. Anesthesiol Pain Med..

[2] Fisher K.S., Arnholt A.T., Douglas M.E. (2009). A randomized trial of the traditional sitting position versus the hamstring stretch position for labor epidural needle placement. Anesth Analg..

[3] Afolayan J.M., Areo P.O., Adegun P.T. (2017). Comparison of ease of induction of spinal anaesthesia in sitting with legs parallel on the table versus traditional sitting position. Pan Afr Med J..

[4] Norris M.C., Barash P., Cullen B.F., Stoelting R.K. (2017). Clinical Anesthesia.

[5] Manggala S.K., Tantri A.R., Satoto D. (2016). Comparison of successful spinal needle placement between crossed-leg sitting position and traditional sitting position in patients undergoing urology surgery. Anesthesiol Pain Med..

[6] Tashayod M.E., Tamadon S. (1980). Spinal block in sitting position without moving the legs. Middle East J Anaesthesiol..

[7] Shaikh F., Brzezinski J., Alexander S. (2013). Ultrasound imaging for lumbar punctures and epidural catheterisations: Systematic review and meta-analysis. BMJ..

[8] Perlas A., Chaparro L.E., Chin K.J. (2016). Lumbar neuraxial ultrasound for spinal and epidural anesthesia: A systematic review and meta-analysis. Reg Anesth Pain Med..

[9] Sebbag I., Tang R., Gunka V. (2018). Effect of table tilt and spine flexion...rotation on the acoustic window of the lumbar spine in pregnant women. Braz J Anesthesiol..

[10] Sandoval M., Shestak W., St..rmann K. (2004). Optimal patient position for lumbar puncture, measured by ultrasonography. Emerg Radiol..

[11] Fisher A., Lupu L., Gurevitz B. (2001). Hip flexion and lumbar puncture: a radiological study. Anaesthesia..

[12] Abo A., Chen L., Johnston P. (2010). Positioning for lumbar puncture in children evaluated by bedside ultrasound. Pediatrics..

[13] Chin K.J., Karmakar M.K., Peng P. (2011). Ultrasonography of the adult thoracic and lumbar spine for central neuraxial blockade. Anesthesiology..

[14] Bilal B., Urfal..o..lu A., ..ks..z G. (2020). Ultrasonographic measurement of the ligamentum flavum at different angles in the lateral tilt position. J Clin Monit Comput..

[15] Arzola C., Davies S., Rofaeel A. (2007). Ultrasound using the transverse approach to the lumbar spine provides reliable landmarks for labor epidurals. Anesth Analg..

[16] Balki M., Lee Y., Halpern S. (2009). Ultrasound imaging of the lumbar spine in the transverse plane: The correlation between estimated and actual depth to the epidural space in obese parturients. Anesth Analg..

[17] Grau T., Leipold R..úW., Horter J. (2001). Paramedian access to the epidural space: The optimum window for ultrasound imaging. J Clin Anesth..

