# Supplementary material for: Rider sitting position widens lumbar intervertebral distance: a prospective observational study
Source: Braz J Anesthesiol. 2021 Apr 19;73(6):758–63. doi: 10.1016/j.bjane.2021.03.010 (PMC10625150; doi:10.1016/j.bjane.2021.03.010)
Supplement: Supplementary file 1 [file mmc1.pdf]

Editing Certificate

This document certifies that the manuscript

The effect of the rider sitting position on the interspinous distance in the lumbar spine in patients undergoing neuraxial anesthesia: a prospective observational study

prepared by the authors

Melike Korkmaz Toker, Basak Altiparmak, Ali Ihsan Uysal, Mustafa Turan, Semra Gumus Demirbilek

was edited for proper English language, grammar, punctuation, spelling, and overall style by one or more of the highly qualified native English speaking editors at AJE.

This certificate was issued on **March 30, 2020** and may be verified on the AJE website using the verification code **05CE-C068-0A07-2F71-5F47**.

Neither the research content nor the authors' intentions were altered in any way during the editing process. Documents receiving this certification should be English-ready for publication; however, the author has the ability to accept or reject our suggestions and changes. To verify the final AJE edited version, please visit our verification page at aje.com/certificate. If you have any questions or concerns about this edited document, please contact AJE at support@aje.com.

AJE provides a range of editing, translation, and manuscript services for researchers and publishers around the world. For more information about our company, services, and partner discounts, please visit aje.com.
